# Metagenomic and Metatranscriptomic Insight Into Oral Biofilms in Periodontitis and Related Systemic Diseases

**DOI:** 10.3389/fmicb.2021.728585

**Published:** 2021-10-13

**Authors:** Yi Huang, Xinyuan Zhao, Li Cui, Shaohong Huang

**Affiliations:** ^1^Stomatological Hospital, Southern Medical University and Guangdong Provincial Stomatological Hospital, Guangzhou, China; ^2^School of Dentistry and Jonsson Comprehensive Cancer Center, University of California, Los Angeles, Los Angeles, CA, United States

**Keywords:** metagenomics, metatranscriptomics, periodontitis, microbial community, systemic diseases

## Abstract

The oral microbiome is one of the most complex microbial communities in the human body and is closely related to oral and systemic health. Dental plaque biofilms are the primary etiologic factor of periodontitis, which is a common chronic oral infectious disease. The interdependencies that exist among the resident microbiota constituents in dental biofilms and the interaction between pathogenic microorganisms and the host lead to the occurrence and progression of periodontitis. Therefore, accurately and comprehensively detecting periodontal organisms and dissecting their corresponding functional activity characteristics are crucial for revealing periodontitis pathogenesis. With the development of metagenomics and metatranscriptomics, the composition and structure of microbial communities as well as the overall functional characteristics of the flora can be fully profiled and revealed. In this review, we will critically examine the currently available metagenomic and metatranscriptomic evidence to bridge the gap between microbial dysbiosis and periodontitis and related systemic diseases.

## Introduction

Periodontitis, triggered by the highly pathogenic biofilm accumulated on teeth, is a chronic inflammatory disease that progressively destroys the supporting periodontal tissues. If left untreated, this oral infectious disease can eventually lead to tooth loss. In addition, accumulating evidence has demonstrated that periodontitis is a potential risk factor for many systemic diseases, such as diabetes, cardiovascular diseases, and rheumatoid arthritis (RA) (Teles et al., 2021). Pathogenic microbes and their products in dental plaque can cause systemic inflammation and immune responses. The prevalence of periodontal diseases is high both in developed and developing countries, ranging from 20 to 50% (Nazir, 2017). In addition, severe periodontitis affects approximately 10% of the global population (Frencken et al., 2017). A survey reported that more than 40% of the U.S. population aged ≥30 years had periodontitis, and 7.8% of these individuals suffered from severe periodontitis (Eke et al., 2020). According to the 4th National Oral Health Epidemiological Survey, periodontitis is highly prevalent in Chinese adults. This issue is especially prevalent in elderly individuals aged 55–74, as 70% of them have detectable periodontal attachment loss, and approximately 15% of them have detectable deep periodontal pockets (≥6 mm). Even among adults aged 35–44, the detection rate of periodontal attachment loss reaches 33%, which demonstrates that periodontitis has a relatively high prevalence even in young adults (Jiao et al., 2021). According to the Global Burden of Disease Study, periodontal disease is a component of the global burden of chronic diseases and therefore represents a significant public health problem (Vos et al., 2017).

It has been well established that polymicrobial biofilms play an essential role in the initiation and progression of periodontitis. However, a number of questions remain to be addressed. For instance, what are the detailed differences in genomic architecture between microbial biofilms from patients with periodontitis and those from healthy volunteers? How do changes in microbial composition drive the pathogenesis of periodontitis? Are there any special microbial features that link to systemic diseases? In recent years, the development of metagenomics and metatranscriptomics has allowed the high-throughput study of microbial communities, which might shed light on the above questions. In this review, we will critically review the currently available evidence to gain a better understanding of the links between microbial dysbiosis and periodontitis and related systemic diseases.

## Overview of Metagenomics and Metatranscriptomics

Metagenomics, the genomic analysis of all microorganisms in the environment, has utilized sequencing analysis to investigate microbial diversity, population structure, evolutionary relationships, functional activity, mutual collaboration, and the association between microorganisms and the environment (Handelsman, 2004). Via high-throughput sequencing, metagenomics is able to produce a significant quantity of sequence data, with between 120 gigabases and 1.5 terabases per run (Quince et al., 2017). The traditional microbial detection and identification approaches are mainly based on traditional culture methods, including sample preparation, broth culture, plate count, isolation of single bacterial colonies and microbial identification, and are not able to detect uncultivated microbial species. The cultivation-independent property and high sequencing efficiency of metagenomic technology make it possible to analyze the functional diversity of microbial communities, interpret the metabolic pathway compositions, explore the interaction patterns between microbes and the environment, and discover featured genes or species with specific functions (Wang and Jia, 2016).

Metatranscriptomics, emerging after metagenomics, mainly studies the gene expression profile and the *in situ* function of microbial communities (Yu et al., 2019), which also avoids the isolation and cultivation of microorganisms and effectively explores biological diversity at deeper and more intricate levels. Metatranscriptomics provides a broader perspective than metagenomics, as it can reveal details about transcriptionally active populations and contribute to exploring the gene expression pattern and functional characteristics of complex microbial communities (Stavros et al., 2016). Through combining metatranscriptomics and metagenomics, the taxonomy and function of the biological community under particular conditions can be linked together, and sophisticated in-depth related functions of the microbial community can be investigated as a whole (Aguiar-Pulido et al., 2016).

**FIGURE 1:**
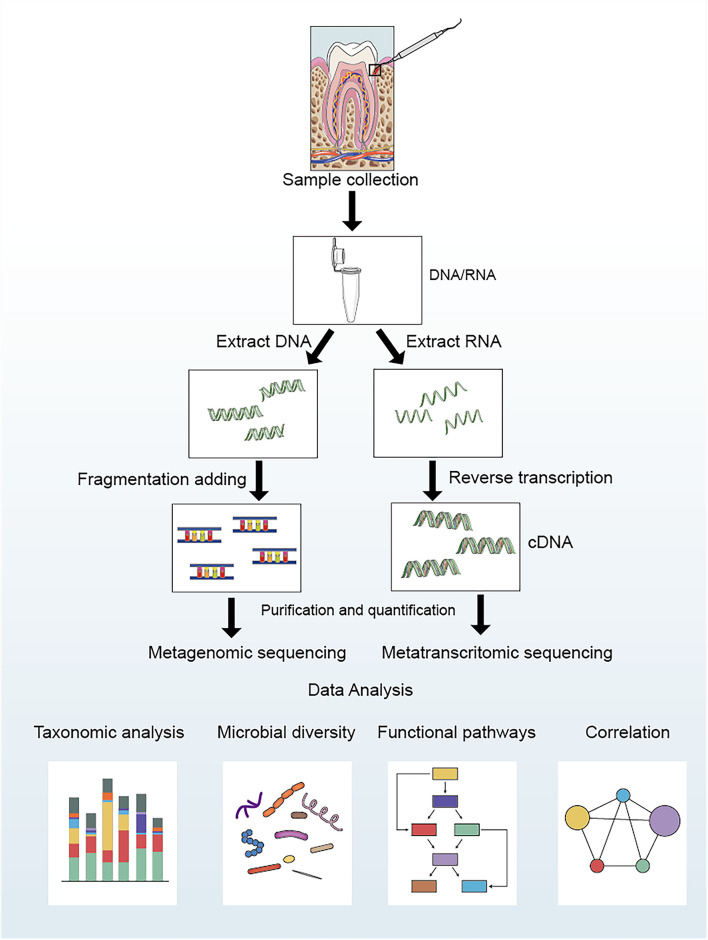
Laboratory flow and bioinformatic analysis for metagenomic and metatranscriptomic studies of oral microbial communities.

The analysis process of metagenomics and metatranscriptomics can be divided into the following four steps (shown in Figure 1): ➀ Sample collection and extraction: according to the purpose and scheme of the experiment, microbial samples are collected from the oral cavity, and DNA/RNA are extracted. ➁ Data acquisition: After extracting the DNA/RNA, the data are obtained by constructing a sequencing library and performing high-throughput sequencing. ➂ Data processing: Quality control should be carried out on the acquired data to remove artificially added primers and low-quality sequences during the process of library construction and sequencing. Simultaneously, it is necessary to compare with the human genome sequence to filter possible human gene contaminants (Davis et al., 2018). Finally, the clean data are compared with multiple databases to determine the species composition and functional composition (Tsute et al., 2010; Escapa et al., 2018). ➃ Statistical analysis: Statistical analysis is conducted based on the sample metadata to explore the potential links between oral microbiota and diseases.

The emergence of next-generation sequencing methods like 454 pyrosequencing, Illumina sequencing technology, ABI SOLiDsequencing technology, and Ion torrent technology as well as third-generation sequencing technology represented by Pacific Biosciences (PacBio) and Oxford Nanopore Technologies (ONT) have put the rapid genome sequencing on the fast track (Slatko et al., 2018). Metagenomics and metatranscriptomics have gradually opened a new chapter in the field of oral microbiology. In 2007, the National Institutes of Health (NIH) launched the Human Microbiome Project (HMP), which sequenced and analyzed microbes in 9 parts of the oral cavity of 242 healthy adults (Huttenhower et al., 2012; Methé et al., 2012). In 2008, the Forsyth Institute and King’s College London Dental Institute jointly established the Human Oral Microbiome Database (HOMD), and subsequent studies have continuously enriched the database resources, which offer a significant reference for studying the role of oral microbes in health and diseases (Dewhirst et al., 2010).

The primary goal of oral microbial metagenomics is to reveal the diversity and screen the functional genes of microbial communities. Belda-Ferre et al. (2012) employed metagenomics sequencing to analyze 8 dental plaque samples from a group of individuals with good oral health, a low-activity caries group (less than 4 cavities) and a high-activity caries group (more than 8 cavities). Significant differences were observed in the microbial communities between healthy and diseased individuals at the taxonomic and functional levels. In addition, dominant bacteria such as *Streptococcus oralis*, *Streptococcus mitis*, and *Streptococcus sanguis* isolated from healthy individuals might be used as probiotics to prevent caries. By metagenomic analysis of dental plaques from individuals with periodontitis and healthy individuals, the microbial community structure was found to be closely related to disease manifestations. Moreover, several functional genes and metabolic pathways related to bacterial chemotaxis and biosynthesis of polysaccharides were overexpressed in periodontitis, suggesting that the formation and alteration of the microbial community in the oral cavity significantly affected the pathogenesis of periodontitis (Wang et al., 2013). Similarly, dramatic difference was observed for the functional potential of the subgingival microbiome from the deep sites in generalized aggressive periodontitis, chronic periodontitis and healthy controls (Altabtbaei, 2016). Faust et al. analyzed the co-occurrence of microbial communities in dental plaque, vagina, gut, and other body site samples from the HMP. The results showed that a small portion of microbes, such as *Firmicutes*, built connections across multiple microbial communities and coordinated microbiota relationships, which verified the potential links between oral diseases and systemic diseases (Faust et al., 2012).

Metatranscriptomics can provide information on the interaction between the functional characteristics of the whole microbial community and diseases, reveal the relevant microbial attributes, and elucidate potential intervention targets. For example, metatranscriptomic analysis of dental caries in children revealed that the alteration of specific enzyme activities might be closely associated with the initiation and progression of caries. The expression level of alcohol dehydrogenase has been found to be increased considerably in a caries-free group, while arginine deiminase and urease levels have been shown to be higher in a dentin caries group (Kressirer et al., 2018). Belstrøm et al. (2017) compared the composition and functional activities of microbial communities in stimulated saliva samples from healthy, periodontitis, and caries individuals with metagenomics and metatranscriptomics. The results showed that the relative abundances of traditional periodontal pathogens such as *Filifactor alocis* and *Porphyromonas gingivalis* and caries-related bacteria such as *Streptococcus mutans* and *Lactobacillus fermentum* were higher in the saliva samples from the periodontitis and caries groups, respectively. In addition, lipid metabolism was found to be suppressed in the caries group, and abnormal carbohydrate metabolism was found in both the periodontitis and caries groups. In brief, the integration of multiomics might be a powerful tool to unravel the complex network of connections between microbial communities and host responses (Manoil et al., 2020).

## Metagenomics and Metatranscriptomics Studies in Periodontitis

### Relationship Between Periodontitis and Oral Microorganisms

**FIGURE 2:**
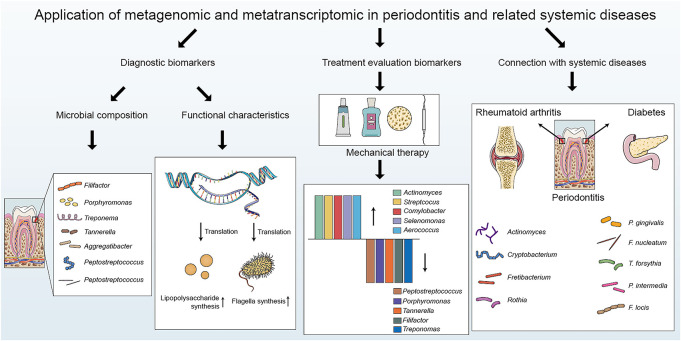
The applications of metagenomics and metatranscriptomics in periodontitis: biomarker detection, treatment evaluation and connection with periodontitis-related systemic diseases.

Saliva and dental plaque can be easily obtained, and thus, they are optimal for investigating the initiation and development of periodontitis (Lasserre et al., 2018). The dynamic balance between the flora and the host is vital for maintaining oral health. Qualitative or quantitative shifts of the oral microbiome lead to microecological dysbiosis, an imbalance responsible for the development of periodontitis (Dewhirst et al., 2010). Under periodontal health circumstances, gram-positive bacteria such as *Actinomyces* and *Streptococcus* dominate the plaque biofilm, while poor oral hygiene results in a shift toward gram-negative bacteria and motile microorganisms (Ezzo and Cutler, 2003). When the microenvironment fluctuates, dysbiosis occurs in the microbial community, resulting in a reduction in species richness and loss of biodiversity (Kumar P. S., 2017). A more dysbiotic microbial community has been observed in well-maintained patients with chronic periodontitis than in healthy individuals (Lu et al., 2020). There is a bilateral impact between the microbial community and the host. An imbalanced microbial community affects the host response, resulting in an inappropriate and uncontrolled level of inflammation, which further damages periodontal tissues (Kilian et al., 2016). The inflammatory response leads to changes in the microbiome and the expression of bacterial virulence factors (Curtis et al., 2020). Metagenomics and metatranscriptomics have provided novel insights into the oral microbiome in periodontitis and related systemic diseases (Figure 2).

### Profiling Periodontitis-Related Pathogens With Metagenomics and Metatranscriptomics

Metagenomic and metatranscriptomic approaches enable the identification of dynamic changes in the composition and structure of the periodontal microflora (Table 1). Detecting the specific and robust bacterial species that cause periodontal microbiota dysbiosis is important not only for understanding the molecular mechanisms of periodontitis initiation and progression but also for providing novel targeted therapy for periodontitis. With the metagenomic method, new microbial species related to periodontitis can be discovered, and the periodontal microbial community can be comprehensively classified. Moreover, metagenomics can clarify the differences in the microbiota between healthy and diseased periodontal tissues, helping confirm dominant periodontal bacteria. By using the 16S rRNA metagenomic approach, six genera, *Filifactor*, *Porphyromonas*, *Treponema*, *Tannerella*, *Aggregatibacter*, and *Peptostreptococcus*, have been found to be significantly enriched in the subgingival plaque samples from severe chronic periodontitis patients compared to those from healthy subjects, indicating that microbial transition might play a crucial role in periodontitis pathogenesis. The metagenomic sequencing targets the entirety of the microbial genetic information, which might help gain a better understanding of the complex microbial communities in periodontal diseases (Tsai et al., 2018). A novel periodontal disease-associated bacterium named *Candidatus Bacteroides Periocalifornicus* (CPB) has been mainly detected in the deep periodontal pockets through metagenomics. It might become a candidate member of the red complex, as its relative abundance has been shown to be significantly associated with that of red complex bacteria and was involved in periodontitis-associated virulence (Torres et al., 2019). Subgingival plaque samples from healthy controls, patients with stable periodontitis and patients with progressing periodontitis have been analyzed by a shotgun metagenomic approach. The study revealed a strong correlation between the loss of oral microbial diversity and periodontitis. In addition, the abundances of *Lactobacillus gasseri* and *Olsenella uli* were increased, while *Campylobacter* was decreased in progressing periodontitis compared to those in stable periodontitis, which indicated that the altered microbial profile might be a potential marker for distinguishing the different periodontitis states (Ai et al., 2017). Shotgun metagenomic analysis of subgingival plaque from chronic periodontitis (CP), localized aggressive periodontitis (LAP) and generalized aggressive periodontitis (GAP) has revealed that genes that regulate acetate-scavenging lifestyle, utilization of alternative nutritional sources, oxidative and nitrosative stress responses, and siderophore production were unique to LAP. In addition, the virulence-associated functionalities and stress response were gradually increased from CP to GAP to LAP (Altabtbaei et al., 2021). Using the whole genome shotgun sequencing method, several overrepresented genes encoding proteins involved in fermentation, antibiotic resistance, adhesion, invasion and intracellular resistance were observed, suggesting a more effective gene-centric methodology to identify biomarkers and periodontitis predictors (Dabdoub et al., 2016). Striking differences in the composition of the subgingival microbiome have also been observed during peri-implantitis development. A total of seven discriminative predominant bacteria, *Porphyromonas gingivalis*, *Tannerella forsythia*, *Porphyromonas endodontalis*, *Fretibacterium fastidiosum*, *Prevotella intermedia*, *Fusobacterium nucleatum*, and *Treponema denticola*, have been identified in peri-implantitis by metagenomic sequencing (Ghensi et al., 2020).

**TABLE 1:** Profiling periodontitis related pathogens with metagenomics and metatranscriptomics.

Microorganism	Sample type	Number of microbial species	Identification method	References
*Filifactor; Porphyromonas; Treponema; Tannerella; Aggregatibacter; Peptostreptococcus*	Subgingival plaque	6 genera	16S rRNA metagenomic approach	Tsai et al., 2018
*Candidatus Bacteroides periocalifornicus*	Subgingival plaque	1 new species	Metagenomic read-classification approach	Torres et al., 2019
*Lactobacillus gasseri; Osenella uli; Campylobacter showae; Atopobium parvulum, etc.*	Subgingival plaque	31 species in stable periodontitis and 34 species in progressing periodontitis	Shotgun metagenomic sequencing approach	Ai et al., 2017
*Aggregatibacter actinomycetemcomitans; Fusobacterium nucleatum; Treponema socranskii; Porphyromonas gingivalis*	Subgingival plaque	65 species in GAP 63 species in LAP	Shotgun metagenomic sequencing approach	Altabtbaei et al., 2021
*Porphyromonas gingivalis; Porphyromonas endodontalis; Tannerella forsythia; Fusobacterium nucleatum; Fretibacterium fastidiosum; Treponema denticola; Prevotella intermedia, etc.*	Subgingival plaque	71 species in the peri-implant microbiome	Shotgun metagenomic sequencing approach	Ghensi et al., 2020
*Porphyromonas gingivalis; Filifactor alocis; Tannerella forsythia; Parvimonas micra, etc.*	Subgingival plaque and saliva	32 species	Metagenomic and metatranscriptomic	Belstrøm et al., 2017
*Porphyromonas; Fusobacterium; Fretibacterium; Filifactor; Parvimonas; Selenomonas; Treponema; Kingella, etc.*	Subgingival plaque	NA	Whole-genome shotgun sequencing approach	Dabdoub et al., 2016
*Fusobacterium nucleatum; Tannerella forsythia; Prevotella tannerae; Porphyromonas gingivalis; Prevotella intermedia*	Subgingival plaque	NA	Metatranscriptomic sequencing approach	Jorth et al., 2014
*Porphyromonas gingivalis; Tannerella forsythia; Treponema denticola*	Subgingival plaque	NA	Metatranscriptomic sequencing approach	Duran-Pinedo et al., 2014
*Tannerella forsythia; Porphyromonas gingivalis*	Subgingival plaque	NA	Metatranscriptomic sequencing approach	Yost et al., 2015

Metatranscriptomics is important for determining the ecosystem function of microbial communities. In addition to clarifying the active microbial community in the disease state, metatranscriptomics also provides information on the functional characteristics of the overall microbial community to explore different functional activities in health or disease conditions. Metatranscriptomic analysis of subgingival plaque samples from patients with aggressive periodontitis has demonstrated that the composition of disease-associated communities varied considerably among patients, whereas the metabolic profiles and virulence gene expression were conserved. For example, the expression levels of genes controlling butyrate production, which promoted the development of periodontitis, increased in microbial communities from different patients, providing more profound insights into the relationship between microbe functional activities and periodontitis (Jorth et al., 2014). Through metatranscriptomic methodology, metabolic activities such as iron acquisition, lipopolysaccharide synthesis, and flagella synthesis have been found to be increased in periodontitis. Surprisingly, most virulence factors whose expression was upregulated in patients with periodontitis were from non-dominant periodontitis pathogens. These findings indicate that metatranscriptomics contributes to enhance the understanding of the core activities that represent the characteristics of periodontitis and the role of individual subgingival organisms in periodontitis (Duran-Pinedo et al., 2014). Cobalamin biosynthesis, proteolysis, and potassium transport have been found to be different in subgingival plaque samples from progressing and stable sites of periodontitis, indicating that changes in microbiome function were closely associated with periodontitis development (Yost et al., 2015).

Most of the studies described above are cross-sectional studies that focused on detecting changes in the composition of microbial communities under different conditions and revealed that microbial dysbiosis is directly related to periodontitis occurrence and development. However, additional longitudinal studies are needed to better understand the correlation between the structural composition/metabolic activities of the periodontitis-associated microbiome and disease progression. In addition, it is necessary to combine the expression profile of the host with microbial community structure, which might contribute to an in-depth understanding of the interaction between the host and microbiome (Solbiati and Frias-Lopez, 2018). Furthermore, proteomics and metabolomics are promising tools for clearly reflecting the functional proteins and final metabolites produced by the microbiome under different conditions, which can accurately reveal the host responses and aid the discovery of potential robust markers at the initial stage of periodontitis.

### Detecting the Alteration of Periodontitis-Related Pathogens With Metagenomics Before and After Treatment

The purpose of initial periodontal therapy is to eliminate pathogenic factors, control inflammation, and curb disease development. Multiomic analysis comparing the changes in periodontal microorganisms before and after initial periodontal therapy is conducive to evaluating therapeutic efficacy (Table 2). Through the metagenomic shotgun sequencing approach, Shi et al. (2015) compared the dynamic changes in the subgingival microbiota in patients with periodontitis pre- and post-periodontal treatment at the same tooth sites. The relative abundance of periodontitis pathogens and disease-associated pathways involving flagella assembly and bacterial chemotaxis were notably changed after periodontal treatment, indicating that the subgingival microbiome composition and functional activities might be potential indicators for disease diagnosis and prognosis prediction. 16S rRNA metagenomic research has been conducted for comparative analysis of subgingival plaque samples from periodontitis patients using antiadhesive hydroxyapatite toothpaste or antimicrobial and antiadhesive fluoride toothpaste following mechanical periodontal therapy. Interestingly, different kinds of toothpaste did not cause significant changes in the microbial composition, suggesting that the toothpaste ingredients had similar effects on the oral microbiota (Hagenfeld et al., 2019). Pyrosequencing of subgingival plaque samples from patients with mandibular class II buccal furcation defects following furcation therapy with enamel matrix derivative (EMD) showed that EMD treatment selectively changed the subgingival microbial composition by reducing the abundance of pathogens and increasing symbiotic flora richness (Queiroz et al., 2017). Califf et al. (2017) simultaneously used 16S rRNA sequencing and shotgun metagenomic sequencing methodologies to perform multiomic analysis on subgingival and supragingival plaque samples from participants who were requested to rinse their mouths with 0.25% sodium hypochlorite or water as controls to compare the differences in the microbial communities in the periodontal pockets. Significant changes in bacterial genera, species, and metabolites were observed before and after treatment, and metabolite richness might be a more sensitive marker for reflecting the changed periodontitis status. Multiomic approaches, including 16S rRNA sequencing, shotgun metagenomics, and metabolomics, have shown that the relative abundance of *Porphyromonas*, *Treponema*, and *Tannerella*, as well as the levels of many metabolites, were decreased following periodontal therapy. Periodontal intervention not only affected the taxonomic composition of subgingival microbial communities but also allowed species benefiting periodontal health to recolonize the oral cavity (Zhang et al., 2020). To the best of our knowledge, very little information is currently available for the metatranscriptomic changes in the oral microbiome following periodontal treatment.

**TABLE 2:** Detecting the alteration of periodontitis related pathogens before and after treatment with metagenomics and metatranscriptomics.

Microorganism that decreased after treatment	Microorganism that increased after treatment	Sample type	Intervention method	Identification method	References
*Peptostreptococcus Porphyromonas Treponema Tannerella Filifactor Olsenella*	*Actinomyces Streptococcus Rothia Bergeyella*	Subgingival plaque	Scaling and root planning (SRP) and oral hygiene instructions	Metagenomic shotgun sequencing approach	Shi et al., 2020
No noticeably different change of alpha and beta diversity before and after periodontal therapy		Buccal/lingual, interproximal, and subgingival plaque	Zinc-substituted carbonated hydroxyapatite toothpaste or amine fluoride/stannous fluoride toothpaste	16S rRNA metagenomic approach	Hagenfeld et al., 2019
*Filifactor alocis Fusobacterium nucleatum Actinomyces Porphyromonas gingivalis*	*Campylobacter Parvimonas Pseudomonas Selenomonas*	Furcation defects plaque	BONE group, EMD group and BONE + EMD group	Pyrosequencing approach	Queiroz et al., 2017
*Porphyromonas Treponema Tannerella Desulfovibrio*	*Streptococcus Aerococcus Slackia Prevotella Selenomonas*	Subgingingival and supragingival plaque	0.25% sodium hypochlorite rinse or water	16S rRNA amplicon sequencing and shotgun metagenomic sequencing approach	Califf et al., 2017

## Evaluation of Periodontitis-Related Systemic Diseases With Metagenomics

In recent years, a growing number of studies have suggested that periodontal pathogens are closely related to the occurrence and development of various systemic diseases, including cardiovascular diseases, diabetes, RA, Alzheimer’s disease, respiratory tract infection, and cancer (Bui et al., 2019). The association between periodontal microbial communities and periodontitis-related systemic diseases detected with metagenomics and metatranscriptomics is summarized in Table 3. The whole metagenomic shotgun sequencing approach has been used to analyze subgingival plaque samples from 4 groups based on the presence/absence of type 2 diabetes and moderate-to-severe periodontitis. The existence of diabetes and/or periodontitis markedly reduced the abundance and diversity of subgingival microorganisms, and an increase in *Anaerolineaceae bacterium* abundance was observed in the patients with both type 2 diabetes and periodontitis (Farina et al., 2019). Saeb et al. (2019) compared the microbial diversity and composition in normoglycemic, impaired glucose tolerance, and diabetes conditions by an ion 16S^TM^ metagenomic sequencing approach. The biological and phylogenetic diversity of oral microbial communities in patients with diabetes and prediabetes was significantly reduced compared with those in patients with normoglycemia. In addition, there was a rise in pathogenic components in the hyperglycemic microbiota. The application of the metagenomic sequencing approach to gingival sulcus biofilms from patients affected by periodontitis and type 2 diabetes revealed that the amount of *Sphingobacteriaceae* bacteria in the biofilm was significantly reduced during periodontitis, and *Sphingobacteriaceae* plays an important role in regulating the sensitivity of cells to insulin, suggesting that periodontitis might significantly affect type 2 diabetes development (Babaev et al., 2017). The metagenomic shotgun sequencing approach was performed for a longitudinal analysis of the subgingival microbiome from patients with periodontitis with/without type 2 diabetes. The results showed that patients with type 2 diabetes were more susceptible to a transformation of the subgingival microbiota to a state of dysbiosis, which might be caused by impaired host metabolism and immune regulation. In addition, a set of microbial genes were found to be enriched in the pathways associated with periodontitis and in the pathways that might connect T2DM and periodontitis. These findings might contribute to a better understanding of the two-way relationship between periodontitis and diabetes (Shi et al., 2020). Metagenomics has also revealed a specific correlation between periodontitis and RA. Significant differences have been found in the subgingival microbial structure in periodontally healthy individuals with or without RA. The relative abundance of gram-negative anaerobes in the subgingival microbial community has been found to be increased in RA, manifesting a state of biological disorder. Arachidonic acid and ester lipid metabolism pathways were enriched in the dental plaque biofilm of RA patients. In addition, the periodontal pathogen *Cryptobacterium curtum* identified in RA generates large amounts of citrulline and subsequently promotes the generation of citrullinated RA autoantigens, which confirms a link between periodontitis and RA (Lopez-Oliva et al., 2018). A longitudinal study investigated fecal, tooth, and salivary samples from individuals with RA and healthy controls by using metagenomic shotgun sequencing. The results revealed that the intestinal and oral microbiomes were consistent, which indicated an overlap of species abundance and function at different body parts. Moreover, the dysbiosis of the oral flora in RA patients was partially reversed after RA treatment. Functionally, the transport and metabolism of iron, sulfur, zinc, and arginine were altered in the microbiota of RA patients. These findings provide novel insights into specific alterations in the intestinal and oral microbiomes of RA patients and suggest a promising method for diagnosis and prognosis prediction based on oral microbiome composition (Zhang et al., 2015). Metagenomic sequencing of supra-gingival dental plaque from patients with Alzheimer’s disease (AD) and healthy controls demonstrated that the microbial diversity was lower in AD. In addition, the numbers of *Lactobacillales*, *Streptococcaceae*, and *Firmicutes/Bacteroidetes* were significantly increased, whereas *Fusobacterium* and Proteobacteria were significantly decreased in patients with AD, indicating that detecting the altered oral microbiota might contribute to the diagnosis of AD in the early stages (Wu et al., 2021). Periodontitis induced-systemic inflammation is a risk factor of cardiovascular disease. Metagenomic shotgun sequencing revealed that the composition of subgingival microbiome varied significantly between severe and mild periodontitis. In addition, the bacteria and metabolic pathways more abundant in severe PD was strongly associated with systemic inflammation, supporting the indirect inflammatory mechanism that the oral microbiome produces inflammatory mediators in the systemic circulation (Plachokova et al., 2021). Metagenomic sequencing of bacterial biofilms revealed that periodontal pathogens *Porphyromonas gingivalis*, *Fusobacterium nucleatum*, *Tannerella forsythia*, and *Treponema denticola* were highly correlated with cytokine IL-1β and vascular flow post sodium nitroprusside treatment, indicating the direct link between the abundance of specific periodontal pathogens and CVD (Kumar P. K. V., 2017). Metagenomic sequencing was performed to compare the microbial communities between HPV+ and HPV− CP granulation-samples. The abundance of *Capnocytophaga ochracea* was increased in HPV + CP samples, while *Porphyromonas endodontalis*, *Macellibacteroides fermentas*, *Treponema phagedenis*, and *Campylo-bacter rectus* species were overexpressed in HPV- CP samples. In addition, the changes in species richness led to activate pathways favoring carcinogenesis, indicating that these species might serve as novel biomarkers for oral cancer (Chowdhry et al., 2019). There is currently little information available on evaluating the connection between oral microbiome and periodontitis related systemic diseases with metatranscriptomics.

**TABLE 3:** Evaluation of periodontitis related systemic diseases with metagenomics and metatranscriptomics.

Microorganism	Systemic diseases	Sample type	Identification method	References
Higher abundance of *Filifactor alocis* and *Treponema* sp. *OMZ 838*	Periodontitis without type 2 diabetics	Subgingival plaque	Whole metagenomic shotgun sequencing approach	Farina et al., 2019
Higher abundance of *Dialister pneumosintes*	Periodontitis with type 2 diabetics			
*Rothia mucilaginosa Prevotella melaninogenica Haemophilus parainfluenzae Streptococcus salivarius*	Diabetes	Subgingival plaque	Ion 16S^TM^ metagenomic sequencing approach	Saeb et al., 2019
*Haemophilus parainfluenzae Rothia mucilaginosa Prevotella melaninogenica*	Impaired glucose tolerance (IGT)			
*Porphyromonas gingivalis Tannerella forsythia Treponema denticola Fusobacterium nucleatum Porphyromonas endodontalis*	Periodontitis with type 2 diabetes	Gingival sulcus	16S metagenomic sequencing approach	Babaev et al., 2017
*Porphyromonas gingivalis Tannerella forsythia Treponema denticola Fusobacterium nucleatum Campylobacter rectus Prevotella intermedia Filifactor alocis*	Periodontitis without type 2 diabetes Periodontitis with type 2 diabetes	Subgingival plaque	Metagenomic shotgun sequencing approach	Shi et al., 2020
*Actinomyces Cryptobacterium Fretibacterium Desulfovibrio Leptotrichia*	Rheumatoid arthritis (RA)	Subgingival plaque	16S rDNA sequencing approach	Lopez-Oliva et al., 2018
*Rothia dentocariosa Lactobacillus salivarius Atopobium* spp. *Cryptobacterium curtum*	Rheumatoid arthritis (RA)	Saliva, tooth and fecal	Metagenomic shotgun sequencing approach	Zhang et al., 2015
*Lactobacillales Streptococcaceae Firmicutes/Bacteroidetes*	Alzheimer’s disease (AD)	Supragingival plaque	PacBio sequencing approach	Wu et al., 2021
*Porphyromonas endodontalis Eubacterium brachy Eubacterium saphenum Propionibacterium propionicum Parvimonas micra*	Cardiovascular disease (CVD)	Subgingival plaque	Metagenomic shotgun sequencing approach	Plachokova et al., 2021
*Porphyromonas gingivalis, Fusobacterium nucleatum, Tannerella forsythia Treponema denticola*	Cardiovascular disease (CVD)	Subgingival plaque	Metagenomic sequencing approach	Kumar P. K. V., 2017
High abundance of *Capnocytophaga ochracea*	HPV infection	Periodontal granulation tissue	Metagenomic sequencing approach	Chowdhry et al., 2019

## The Limitation of the Metagenomics and Metatranscriptomics

The consensus findings of metagenomic and metatranscriptomic studies in periodontitis and related systemic diseases have been summarized in Table 4. One major shortcoming of current studies is that the sample size is small, which might significantly affect the interpretation of the impact of periodontal treatment on the periodontal microbiome composition. In addition, the study design, research object, sampling method, therapeutic strategy, and duration of follow-up are very heterogeneous. Therefore, it is still unclear whether metagenomics and metatranscriptomics are more promising than traditional clinical indicators for evaluating the effectiveness of periodontal treatment (Feres et al., 2021). Additional well-designed randomized clinical trials with longer follow-up times, combined with multiomic analysis, are warranted to confirm whether metagenomic/metatranscriptomic changes can be used as outcome indicators and contribute to personalized periodontal therapy.

**TABLE 4:** Consensus findings of metagenomic and metatranscriptomic studies in periodontitis and related systemic diseases.

Identification method	Sample type	Periodontal condition	Systemic condition	Periodontal intervention	Findings	References
Metagenomic	Subgingival plaque and saliva	Periodontitis	Health	NA	Increased abundance of *Porphyromonas, Filifactor, Treponema and Tannerella*	Belstrøm et al., 2016; Dabdoub et al., 2016; Ai et al., 2017; Tsai et al., 2018; Ghensi et al., 2020
Metatranscriptomic	Subgingival plaque	Periodontitis	Health	NA	Increased abundance of *Porphyromonas gingivalis and Tannerella forsythia*	Duran-Pinedo et al., 2014; Jorth et al., 2014; Yost et al., 2015
Metagenomic	Subgingingival/supragingival plaque	Periodontitis	Health	Non-surgical periodontal treatment	Common decreased abundance of *Porphyromonas, Treponema*, and *Tannerella*, increased abundance of *Selenomonas* after non-surgical periodontal treatment	Shi et al., 2015; Califf et al., 2017; Queiroz et al., 2017; Hagenfeld et al., 2019
Metagenomic	Subgingival and supragingival plaque, gingival sulcus and saliva	Periodontitis	Disease	NA	1) The abundance and diversity of oral microorganisms in individuals with periodontitis and systemic diseases were significantly decreased; 2) The oral microbial community in patients with systemic diseases are more likely to display a state of dysbiosis; 3) Periodontal pathogens and related metabolic pathways are strongly corelated with systemic diseases, serving as potential biomarkers.	Babaev et al., 2017; Kumar P. K. V., 2017; Lopez-Oliva et al., 2018; Chowdhry et al., 2019; Farina et al., 2019; Saeb et al., 2019; Shi et al., 2020; Zhang et al., 2020, Wu et al., 2021; Plachokova et al., 2021

## Monitoring the Onset and Progression of Periodontitis With Proteomics and Metabolomics

Proteomics and metabolomics are powerful tools for discovering novel and robust disease markers. In addition, the disease-specific proteins or metabolites can be effortless detected in gingival crevicular fluid (GCF), saliva and dental plaque due to their easy accessibility and availability. Therefore, proteomics and metabolomics have already shown great promise for monitoring the onset and progression of periodontitis. Proteomics analysis of saliva samples from patients with periodontitis, patients with dental caries and healthy individuals revealed that the levels of complement proteins and inflammatory mediators were increased in periodontitis and dental caries (Belstrøm et al., 2016). Similarly, the proteomics analysis showed that beta-2-glycoprotein I, α-fibrinogen, hemopexin, and plasminogen were abnormally expressed in the saliva samples from patients with periodontitis compared to the healthy controls (Orti et al., 2018). Bostanci et al. (2018) compared the protein profiles from unstimulated saliva samples of periodontitis patients, gingivitis patients and healthy subjects. The results demonstrated that metalloproteinase-9, ras-related protein-1 and actin-related protein 2/3 complex subunit 5 were significantly upregulated, while clusterin and deleted in malignant brain tumors 1 were downregulated in the periodontitis group compared to the control group. Interestingly, by comparing the proteomic profiles of pre- and post-treatment GCF samples, a robust protein signature including azurocidin, lysozyme C, myosin 9, and smooth muscle actin was built up for determining the clinical endpoints for CP (Guzman et al., 2018). Similar to proteomics, metabolomics has also been widely used for the diagnosis and prognosis prediction of periodontitis. Metabolomics analysis of GCF samples of patients with general chronic periodontitis and healthy controls indicated that the levels of citramalic acid and N-carbamylglutamate showed good performance for diagnosis of general chronic periodontitis (Pei et al., 2020). The metabolomic profiles of serum and GCF samples varied significantly between the patients with aggressive periodontitis and healthy subjects. Specifically, the levels of urea and allo-inositol were increased, while glutathione, 2,5-dihydroxybenzaldehyde, adipic acid and 2-deoxyguanosine were decreased in the serum samples from patients with aggressive periodontitis. For the GCF samples, the levels of noradrenaline, uridine, α-tocopherol, dehydroascorbic acid, xanthine, galactose, glucose-1-phosphate, and ribulose-5-phosphate were higher in patients with aggressive periodontitis, while thymidine, glutathione and ribose-5-phosphate were lower in the disease group (Chen et al., 2018). By integrating 16S rRNA metagenomic sequencing and metabolomics, many abnormally expressed metabolites were found to be positively correlated with periodontal pathogens such as *Porphyromonas, Prevotella*, and *Fusobacterium* species (Na et al., 2021). Similarly, a strong correlation was found between lipid mediators and subgingival microbiome profile, suggesting that inflammation induced by lipid mediators significantly affected the microbial composition in periodontitis (Lee et al., 2021). Metabolomics might also be used for evaluating the therapeutic efficacy. The salivary metabolomic profile of patients with generalized periodontitis was markedly altered by non-surgical periodontal therapy. Interestingly, many important metabolites returned to normal levels following treatment (Citterio et al., 2020). Interestingly, a recent multiomics study showed that the levels of hosphatidylcholines, plasmenyl-phosphatidylcholines, ceramides, and host proteins related to actin filament rearrangement were increased in the dental plaques from patients with periodontal diseases compared to the healthy controls (Overmyer et al., 2021). Therefore, efficiently integrating proteomics/metabolomics with metagenomics/metatranscriptomics might provide a more comprehensive understanding of the interaction between oral microbiome and periodontitis.

## Future Perspective and Conclusion

Collectively, the composition and structure of periodontal microbial communities based on different levels of periodontal microecological bacterial taxonomy as well as the functional activity characteristics can be determined with metagenomics and metatranscriptomics. In addition, exploring the potential correlation between the dynamic changes in the functional behaviors of the periodontal microbiome and the progression and outcome of periodontitis provides valuable information for developing effective prevention and treatment strategies. However, a high degree of host interference, low sensitivity in detecting low-abundance genes, and high detection cost are the limitations of metagenomics. Furthermore, metatranscriptomics is not able to detect the final metabolites of the microbial communities. Therefore, more powerful evidence-based experimental studies are needed. Combining proteomic and metabolomic analyses is vital to reflect the functional proteins and metabolites produced by the microbiome under different conditions. The integration of various microbial detection methodologies will contribute significantly to our understanding of the complex subgingival microbial community and periodontitis pathogenesis.

## Author Contributions

SH and LC: conceptualization and funding acquisition. YH, XZ, SH, and LC: writing–original draft and editing. All authors contributed to the article and approved the submitted version.

## Conflict of Interest

The authors declare that the research was conducted in the absence of any commercial or financial relationships that could be construed as a potential conflict of interest.

## Publisher’s Note

All claims expressed in this article are solely those of the authors and do not necessarily represent those of their affiliated organizations, or those of the publisher, the editors and the reviewers. Any product that may be evaluated in this article, or claim that may be made by its manufacturer, is not guaranteed or endorsed by the publisher.
